# Acute inflammation and psychomotor slowing: Experimental assessment using lipopolysaccharide administration in healthy humans

**DOI:** 10.1016/j.bbih.2020.100130

**Published:** 2020-08-20

**Authors:** Analena Handke, John Axelsson, Sven Benson, Karoline Boy, Vera Weskamp, Till Hasenberg, Miriam Remy, Johannes Hebebrand, Manuel Föcker, Alexandra Brinkhoff, Meike Unteroberdörster, Harald Engler, Manfred Schedlowski, Julie Lasselin

**Affiliations:** a Institute of Medical Psychology and Behavioral Immunobiology, University Hospital Essen, Essen, Germany; b Stress Research Institute, Stockholm University, Stockholm, Sweden; c Division for Psychology, Department of Clinical Neuroscience, Karolinska Institutet, Stockholm, Sweden; d Helios Adipositas Zentrum West, Helios St. Elisabeth Klinik Oberhausen, Witten/Herdecke University, Oberhausen, Germany; e Department of Child and Adolescent Psychiatry, Psychosomatics and Psychotherapy, University Hospital Essen, University of Duisburg-Essen, Germany; f Department of Child and Adolescent Psychiatry, Psychosomatics and Psychotherapy, University Hospital Muenster, Muenster, Germany; g Department of Nephrology, University Hospital Essen, Essen, Germany; h Department of Neurosurgery, University Hospital Essen, Essen, Germany; i Osher Center for Integrative Medicine, Department of Clinical Neuroscience, Karolinska Institutet, Stockholm, Sweden

**Keywords:** Inflammation, Psychomotor slowing, Reaction time, Go/no-go, Sickness, Lipopolysaccharide, Cytokine

## Abstract

Data from clinical and cross-sectional studies suggest that inflammation contributes to psychomotor slowing and attentional deficits found in depressive disorder. However, experimental evidence is still lacking. The aim of this study was to clarify the effect of inflammation on psychomotor slowing using an experimental and acute model of inflammation, in which twenty-two healthy volunteers received an intravenous injection of lipopolysaccharide (LPS, dose: 0.8 ​ng/kg body weight) and of placebo, in a randomized order following a double-blind within-subject crossover design. A reaction time test and a go/no-go test were conducted 3 ​h after the LPS/placebo injection and interleukin (IL)-6 and tumor necrosis factor (TNF)-α concentrations were assessed. No effect of experimental inflammation on reaction times or errors for either test was found. However, inflammation was related to worse self-rated performance and lower effort put in the tasks. Exploratory analyses indicated that reaction time fluctuated more over time during acute inflammation. These data indicate that acute inflammation has only modest effects on psychomotor speed and attention in healthy subjects objectively, but alters the subjective evaluation of test performance. Increased variability in reaction time might be the first objective sign of altered psychomotor ability and would merit further investigation.

## Highlights

• The effect of experimental inflammation on psychomotor slowing was assessed.
• Acute inflammation does not affect psychomotor speed in two reaction time tasks.
• Inflammation altered the subjective evaluation of test performance.
• In exploratory analyses, the variability of reaction time over time was affected.

## 1 Introduction

Psychomotor retardation is characterized by a slowing-down of physical movements, emotions, and thoughts (Caligiuri et al., 2003). Psychomotor retardation can be objectively measured by psychomotor slowing, which includes a mental component (slower reaction time) and a physical component (motor slowing). Psychomotor slowing is a core symptom of depression according to the diagnostic and statistical manual of mental disorders (DSM-5) (American Psychiatric Association, 2013; Davidson et al., 1985) that is relatively resistant to anti-depressive first-line treatments, including selective serotonin reuptake inhibitors (Taylor et al., 2006). It is therefore important to achieve a better understanding of the mechanisms underlying this symptom (Berlim et al., 2020; Culmsee et al., 2019).

Peripheral inflammatory processes are hypothesized to be one of the mechanisms underlying psychomotor slowing. Cytokines that are released during acute inflammation are able to modulate neurotransmitter and neuroendocrine systems in the central nervous system, and to induce an array of behavioural changes called sickness behaviour, including psychomotor slowing (Brydon et al., 2008; Capuron et al., 2012; Dantzer et al., 2008; Felger, 2017; Felger and Miller, 2012). Clinical studies in hepatitis C patients undergoing cytokine therapy found only alterations at the motor level (decreased motor speed), but not at the reaction time level (mental level), both in a simple reaction time task and in a five-choice task (Haroon et al., 2015; Majer et al., 2008). Levels of inflammatory markers were also found to relate to slowing of motor speed, but not to reaction time, in patients suffering from major depressive disorder (Goldsmith et al., 2016). A few experimental inflammation studies have reported slower reaction times during inflammation, but only for complex cognitive tasks, i.e. the Stroop task (Brydon et al., 2008; Lyall et al., 2019; Nicoletti et al., 2004) and the n-back test (Grigoleit et al., 2011), which involve several cognitive functions, such as cognitive flexibility and working memory, as well as the integration of processing in several brain areas, and thus a high cognitive load. The effect of experimental inflammation, in particular of the model of lipopolysaccharide (LPS) administration, on reaction times in pure tasks of psychomotor slowing is unclear. The model of LPS administration in healthy humans has been extensively used over the past 30 years to understand the mechanisms underlying cytokine-induced behavioral changes, because of its relevance for inflammation-associated depression (DellaGioia and Hannestad, 2010; Lasselin et al., 2020b, in press; Schedlowski et al., 2014). This model allows assessing the behavioral effects of inflammation in a safe and highly standardized manner, but the characterization of its effects on psychomotor slowing is lacking.

The main aim of this study was to assess the effect of the administration of LPS on reaction time using a simple reaction time test and a higher demand test, the go/no-go test. We hypothesized that subjects would exhibit slower reaction times under acute systemic inflammation, and in particular in the test with higher cognitive demand, i.e., the go/no-go test, also measuring inhibition. Additionally, we hypothesized that higher LPS-induced plasma cytokine levels will relate to slower reaction time. We also assessed, in an exploratory way, the variability of reaction time over time during the test, as the effect of inflammation could result in difficulties to maintain performance, something that would result in an increased variability throughout the whole test, as seen after sleep loss (Zhou et al., 2011).

## 2 Methods

This study is part of a larger study assessing the immune and behavioral effects of LPS administration in obese *versus* normal-weight subjects (Lasselin et al., 2020a). For the current study, only the normal-weight subjects were included. Details on the protocol have been given previously (Lasselin et al., 2020a) and are briefly summarized herein. The study was approved by the local ethics review board of the University of Duisburg-Essen (reference number: 15-6503-BO).

### 2.1 Participants

Twenty-five healthy normal-weight subjects participated in this within-subjects study. Only healthy volunteers aged 18–35 years with a normal body mass index (between 18.5 and 25 ​kg/m^2^) were included. Exclusion criteria were: excessive sport or alcohol consumption, smoking, pregnancy, diagnosed physiological or psychiatric disease, abnormal blood analyses, regular medication intake (except contraception for women), and infectious episode within the last two weeks. All females had to be under contraceptional therapy for participating in the study. Participants received a written and oral description of the study before provided a written informed consent and were remunerated 310–360 euros.

Among the 25 participants, three individuals were excluded *a priori* based on the defined exclusion criteria: one subject was excluded because of high values (i.e. >mean+3SD) in the baseline Beck Depression Inventory (BDI-II) score at one of the study sessions, and two other subjects were excluded because of high baseline values in cytokines (i.e. >mean+5SD), suggesting a possible ongoing infection. Thus, 22 individuals were included into data analysis (age: 24.7 ​± ​3.5 years; 13 (59%) women). Of note, similar results were found were including the 25 individuals (see Table S1).

### 2.2 Experimental design

A double blind, crossover, placebo-controlled design was used. The volunteers participated in two study sessions, and received an intravenous injection of either LPS (Reference Standard Endotoxin from *Escherichia coli*, lot H0K354, United States Pharmacopeia, Rockville, MD, USA) at 0.8 ​ng/kg body weight, or placebo (i.e. physiological saline) in a randomized order, with at least one week of wash-out. The LPS had been subjected to a microbial safety testing routine by the German Federal Agency for Sera and Vaccines (Paul-Ehrlich Institute, Langen, Germany) and was stored at −20 ​°C until use. LPS/Placebo injection was done in the morning, between 8.45 a.m and 10.30 a.m. During each study session, blood samples were taken before and 1 ​h, 2 ​h, 3 ​h, 4 ​h, 6 ​h, 24 ​h after the injection.

### 2.3 Reaction time tests

On both study days, the simple reaction time and go/no-go tests were conducted 3 ​h after the injection (just after the peak of the peripheral inflammatory response, see Fig. S1), after blood sampling and the completion of the questionnaires, sitting in bed in the quiet hospital room. The tests, chosen from the WakeAPP test battery, were provided using a computerized application on an iPad® (Holding et al., 2017). In the simple reaction time test, subjects had to press a button on the screen, using their dominant hand, as fast as possible when the letter “p” appeared on the screen. In the go/no-go test, subjects had to press a button on the screen, using their dominant hand, as fast as possible when the letter “b” appeared on the screen, but *not* when the letter “p” appeared on the screen. The tests were given in a randomized order and each test lasted 3 ​min. Reaction time, the percentage of errors, and standard deviation of reaction time were obtained. When each of the tests was finished, subjects had to rate two questions on a 9-level scale about self-rated performance (“Wie haben Sie in dem Test abgeschnitten?” ​= ​“How did you perform at the test?”, from “very poor” to “very good”) and effort spent in the test (“Wie viel Mühe haben Sie in den Test investiert?” ​= ​“How much of an effort did you make to complete the test ?”, from “no effort” to “maximum effort”).

Data for both tests was missing in one subject in the placebo condition due to technical issue. Furthermore, data from the go/no-go test from two subjects (one in the LPS condition, one in the placebo condition) was not used because of suspected misunderstanding of the instructions (as indicated by 95% of errors made in the test).

### 2.4 Inflammatory cytokines

Plasma concentrations of TNF-α and IL-6 were measured using a high-sensitivity multiplex assay (Human Mag Luminex Performance AssayRnD Systems, MN, USA) on a Luminex 200 System (software xPONENT 3.1, Luminex corporation, Austin, Texas) according to the manufacturer’s protocol. The minimum detectable dose (MDD) of the assay was 0.28 ​pg/mL for IL-6 and 0.58 ​pg/mL for TNF-α. IL-10 was also measured but not used in the current study.

### 2.5 Statistics

Statistical analyses were conducted using SPSS Statistics 25 using a significant p-value set at p ​< ​.05.

The effect of LPS *versus* placebo administration on concentrations of TNF-α and IL-6 was assessed using mixed linear models (MLM), with condition (LPS/placebo), time, and condition x time as fixed effects, subjects’ ID as random effect and study session and time as repeated variables.

MLM for the continuous outcomes (reaction time, percentage of errors, standard deviation) and generalized estimated equations (GEE) (ordinal logistic) for the ordinal subjective outcomes (self-rated performance and effort) were used to assess the effect of LPS *versus* placebo administration on the outcomes of the two reaction time tests, as well as the associations between cytokine concentrations and reaction time tests outcomes. Study session and test order were entered as repeated variables. A random intercept was used in the MLM. Percentage of errors and standard deviation were log-transformed to rectify skewness and kurtosis. The association between cytokine concentrations and percentage of errors was not assessed because of the low frequency of errors (1–6% in average). Peaks of cytokine concentrations were used, as previously accomplished (Lasselin et al., 2017), as they represent the largest effect of LPS administration and reflect how much cytokines would have had an impact on the brain. Peaks IL-6 and TNF-α were measured 2 ​h after LPS administration for the majority (68% and 77%, respectively) of participants. Peak IL-6 concentrations were log-transformed to rectify skewness and kurtosis.

## 3 Results

LPS administration induced significant and transient increases in plasma concentrations of TNF-α and IL-6 (Fig. S1 and Table S2).

### 3.1 Reaction time tests outcomes after LPS versus placebo administration

**Fig. 1:**
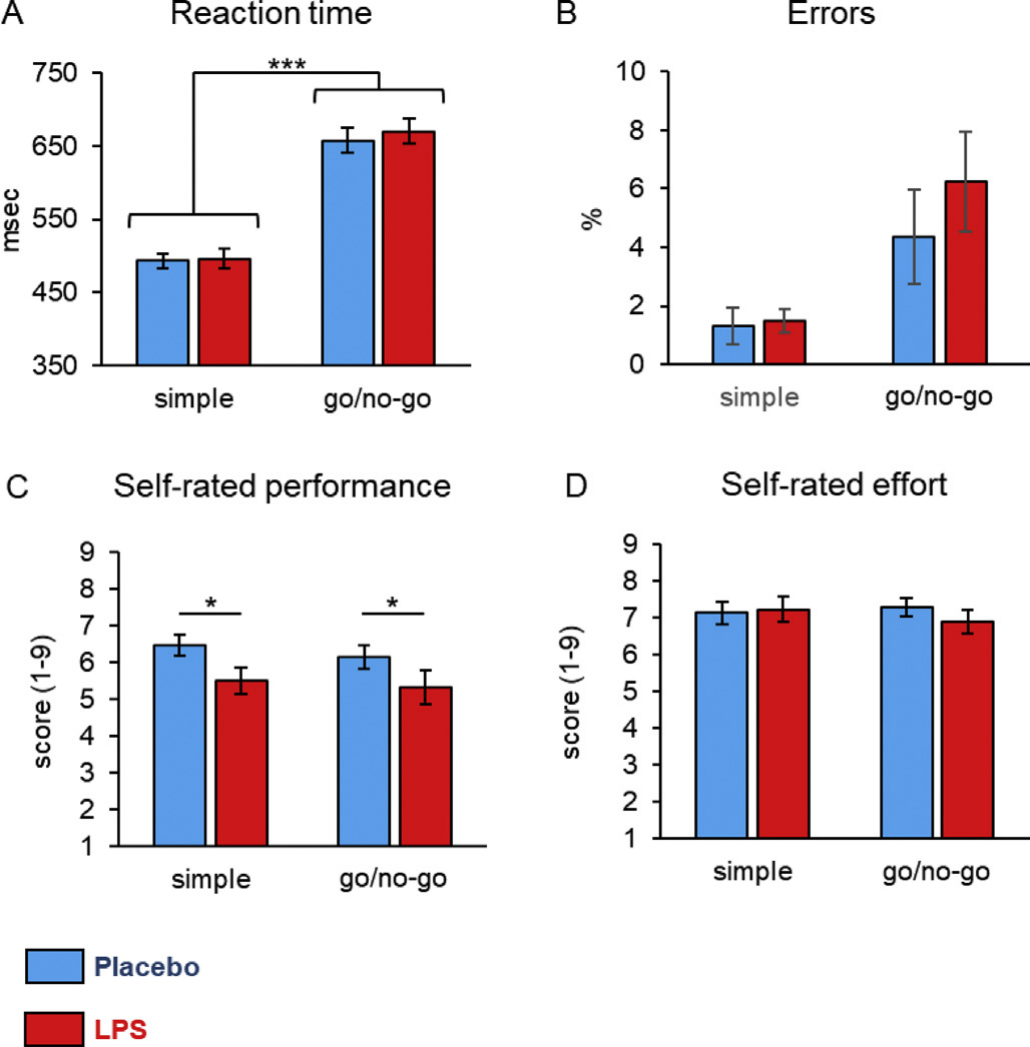
**Effect of LPS administration (0.8 ​ng/kg body weight)vs placeboon reaction timetests outcomes 3h after the injection.** Higher rating score (1–9) indicates better self-rated performance and more effort put in the task. See text for detailed statistics. ∗∗∗p ​< ​.001, ∗p ​< ​.05.

Reaction time was significantly slower in the go/no-go test compared to the simple reaction time test (*b*(SD) ​= ​159.07(13.01), p ​< ​.001), with no significant main effect of treatment (*b*(SD) ​= ​9.38(13.48), p ​= ​.490) and no significant interaction effect (*b*(SD) ​= ​6.51(18.66), p ​= ​.729), indicating no significant difference in reaction time after LPS administration compared to placebo administration in both tests (Fig. 1A). Errors were very few (1–6% in average) and did not differ significantly between the two tests and conditions (go/no-go: *b*(SD) ​= ​0.11(0.13), p ​= ​.390; LPS: *b*(SD) ​= ​0.06(0.12), p ​= ​.638; interaction: *b*(SD) ​= ​0.17(0.17), p ​= ​.327) (Fig. 1B). A significant interaction was found for reaction time variability (go/no-go: *b*(SD) ​= ​-0.02(0.06), p ​= ​.737; LPS: *b*(SD) ​= ​-0.09(0.06), p ​= ​.132; interaction: *b*(SD) ​= ​0.20(0.08), p ​= ​.021), indicating that reaction time fluctuated more after LPS administration compared to placebo administration in the go/no-go test (Fig. S2).

### 3.2 Self-rated performance and effort after LPS versus placebo administration

Participants rated their performance lower after LPS administration (*b*(SD) ​= ​-0.94(0.40), p ​= ​.018), independently of the type of task (go/no-go: *b*(SD) ​= ​-0.30(0.32), p ​= ​.341; interaction *b*(SD) ​= ​0.01(0.49), p ​= ​.982) (Fig. 1C). Finally, there was no significant difference in how participants rated their effort used in the tasks in both tests and in both conditions (go/no-go: *b*(SD) ​= ​0.05(0.24), p ​= ​.837; LPS: *b*(SD) ​= ​0.20(0.36), p ​= ​.589; interaction: *b*(SD) ​= ​-0.50(0.34), p ​= ​.140) (Fig. 1D).

### 3.3 Associations between cytokine concentration and reaction time tests outcomes after LPS administration

No significant associations between IL-6 or TNF-α peak concentrations with reaction time (IL-6 log: *b*(SD) ​= ​-0.50(42.81), p ​= ​.252; TNF-α: *b*(SD) ​= ​-0.003(0.14), p ​= ​.985) and with reaction time variability were found (IL-6 log: *b*(SD) ​= ​-0.24(0.13), p ​= ​.076; TNF-α: *b*(SD) ​= ​-0.0002(0.0004), p ​= ​.574). However, there was a significant negative association between IL-6 peak concentrations and self-rated effort put in the task (log, *b*(SD) ​= ​-2.49(1.20), p ​= ​.038). Furthermore, peak concentrations of TNF-α were negatively associated with self-rated performance (*b*(SD) ​= ​-0.009(0.004), p ​= ​.020). There were no significant associations between IL-6 concentrations and self-rated performance (*b*(SD) ​= ​1.05(1.33), p ​= ​.429), and between TNF-α concentrations and self-rated effort (*b*(SD) ​= ​0.004(0.005), p ​= ​.420).

## 4 Discussion

Despite a pronounced LPS-induced acute inflammatory response with substantially elevated cytokine levels, we found no evidence for inflammation-associated effects on psychomotor reaction times in tests that do not involve higher cognitive functions or complex motor behaviour. The results are in line with previous studies in clinical populations reporting a significant *motor* slowing in association with inflammation, but no clear *mental* slowing (Goldsmith et al., 2016; Haroon et al., 2015; Majer et al., 2008). This raises the question regarding inflammation as the mechanism underlying mental slowing in depressed patients (Goldsmith et al., 2016).

Our findings indicate possible metacognitive alterations associated with inflammation during reaction time tests. Subjects rated their performance worse in the LPS condition compared to the placebo condition, and higher LPS-induced concentrations of cytokines were associated with worse perceived performance and lower self-rated effort. This suggests that, despite the apparent absence of effect of inflammation on psychomotor slowing, individuals feel like they perform less well and have more difficulties to put effort in the tasks. This finding is in line with the notion that acute inflammation favours a negative bias (Benson et al., 2017), according to an augmented effect of sad mood on affective cognition via information processing (Benson et al., 2017), and decreases self-esteem (Kotulla et al., 2018). This negative self-perception, self-doubt, and low self-esteem could facilitate the development of depression (Sowislo and Orth, 2013).

Speculatively, it is also possible that inflammation induces central effects during the reaction time tests, but that compensatory mechanisms help maintain objective performance. A previous study assessing visuospatial attention in hepatitis C patients under immunotherapy has suggested the notion of compensatory mechanisms during inflammation (Capuron et al., 2005). In this study, patients under immunotherapy had similar reaction time and performance accuracy than control patients, but stronger brain activation in a brain structure central for cognitively demanding tasks, the anterior cingulate cortex. The authors thus suggested that stronger activation of this structure could allow maintaining objective performance during inflammation (Capuron et al., 2005). This would however induce a greater cognitive load, which might be reflected in a feeling of performing worse and more difficulties to put effort or the perception of expending more effort in a cognitive task. As cognitive demand increases, however, compensatory mechanisms could fail. Arguably, the current findings that inflammation is associated with worse perceived performance and lower self-rated effort could indicate a greater cognitive load to maintain similar performance, but this notion should be verified by assessing cognitive processes with brain imaging along with behavioral performance in future studies. This notion is however supported by our exploratory analyses on reaction time variability, which showed that the performance in the go/no-go task was fluctuating significantly more after LPS administration compared to placebo. Increased variability might indicate a difficulty to maintain performance throughout the whole task, and might be the first objective sign of altered psychomotor ability. When a higher effort is required, increased variability would also translate into general attentional alterations, such as observed after sleep loss (Basner et al., 2013). This would explain why slower reaction time was found to be objectively altered during experimental inflammation only in tasks with higher attentional demand, such as the Stroop task and the n-back test (Brydon et al., 2008; Grigoleit et al., 2011; Nicoletti et al., 2004). It is also possible that such compensatory mechanisms would fail when inflammation becomes chronic, such as in depression, or when inflammation interacts with other comorbidities.

The main limitation lies in the rather small sample size. However, LPS administration induced a robust inflammatory response and clear sickness symptoms, negative mood, fatigue, and anxiety symptoms (Lasselin et al., 2020a). In addition, smaller sample sizes have been used in previous studies with sufficient statistical power to observe a significant effect of acute inflammation on cognitive functioning (Brydon et al., 2008; Grigoleit et al., 2011). While the present study cannot make conclusions of the existence of small-to mid-sized effect sizes, it clearly shows that inflammation does not result in a strong psychomotor slowing. It is also possible that no effect was observed because of the time-point chosen to perform the reaction time tests, i.e. 3 ​h after injection. This time point was chosen because of organizational reasons (other tests were performed before). However, this time point is an established time point to assess the behavioral effects of cytokines (Lasselin et al., 2016, 2020b; Cho et al., 2016; Engler et al., 2017; Lasselin et al., in press). Furthermore, the reaction time tests were rather short. Longer tests would have demanded more effort and might have reduced the ability to compensate for the effect of inflammation. While longer reaction time tests are often used, 3-min tasks are sensitive to fatigue (Basner et al., 2011), and the purpose in the present study was not to measure fatigue-induced lapses but rather ability to keep up reaction times.

In conclusion, this study indicates no objective psychomotor slowing during acute inflammation, except for slight changes of the variability of reaction time over time during the task. Signs of subjective effects of inflammation were nonetheless observed, with individuals perceiving performing less well and having more difficulties in putting effort in the cognitive tasks during inflammation.

## Funding

The study was supported by the 10.13039/100003579Alexander von Humboldt foundation (Germany, Humboldt fellowship for postdoctoral researchers) [grand number 1156790 to JL].

## Data sharing

Dataset of this study is available at https://osf.io/jqs2e/?view_only=bda3bdc253104c7fad3472b30dd7ba3c.

## Declaration of competing interest

The authors declare no conflict of interest.
