# Supplementary material for: Acute inflammation and psychomotor slowing: Experimental assessment using lipopolysaccharide administration in healthy humans
Source: Brain Behav Immun Health. 2020 Aug 20;8:100130. doi: 10.1016/j.bbih.2020.100130 (PMC8474655; doi:10.1016/j.bbih.2020.100130)

**Handke et al. Supplementary data.**

**Table S1. Comparison of the findings with the entire population (n=25) and excluding the three individuals based on the defined exclusion criteria.**

| Results: | |
| --- | --- |
| N=25 | N=22 (current results) |
| - Reaction time:  go/no-go vs simple: b(SD)=149.21(13.15), p<.001  LPS vs placebo: b(SD)=-5.72(19.43), p=.770  interaction: b(SD)=6.37(20.01), p=.753  - Errors:  **go/no-go vs simple: b(SD)=.27(.12), p=.024**  LPS vs placebo: b(SD)=.14(.11), p=.222  interaction: b(SD)=.01 (.16), p=.962  - Reaction time variability:  go/no-go vs simple: b(SD)=-.05(.07), p=.434  LPS vs placebo: b(SD)=-.01(.06), p=.867  **interaction: b(SD)=.13(.09), p=.148**  - Self-rated performance:  go/no-go vs simple: b(SD)=-.30(.29), p=.313  LPS vs placebo: b(SD)=-.91(.37), p=.014  Interaction: b(SD)=.03(.45), p=.949  - Self-rated effort:  go/no-go vs simple: b(SD)=.04(.22), p=.843  LPS vs placebo: b(SD)=.18(.36), p=.622  interaction: b(SD)=-.41(.32), p=.202  - Associations with cytokine concentrations  IL-6 log-reaction time: b(SD)=7.68(52.25), p=.885  TNF-α-reaction time: b(SD)=-.10(.17), p=.577  IL-6 log-reaction time variability:  b(SD)=-2.35(27.25), p=.933  TNF-α-reaction time variability:  b(SD)=-.01(.09), p=.910  IL-6 log-self-rated performance:  b(SD)=.72(1.21), p=.550  TNF-α-self-rated performance:  b(SD)=-.009(.004), p=.011.  IL-6 log-self-rated effort:  b(SD)=-2.22(1.15), p=.054  TNF-α-self-rated effort:  b(SD)=.002(.004), p=.604. | - Reaction time:  go/no-go vs simple: b(SD)=159.07(13.01), p<.001  LPS vs placebo: b(SD)=9.38(13.48), p=.490  interaction: b(SD)=6.51(18.66), p=.729  - Errors:  **go/no-go vs simple: b(SD)=.11(.13), p=.390**  LPS vs placebo: b(SD)=.06(.12), p=.638  interaction: b(SD)=.17(.17), p=.327  - Reaction time variability:  go/no-go vs simple: b(SD)=-.02(.06), p=.737  LPS vs placebo: b(SD)=-.09(.06), p=.132  **interaction: b(SD)=.20(.08), p=.021**  - Self-rated performance:  go/no-go vs simple: b(SD)=-.30(.32), p=.341  LPS vs placebo: b(SD)=-.94(.40), p=.018  Interaction: b(SD)=.01(.49), p=.982  - Self-rated effort:  go/no-go vs simple: b(SD)=.05(.24), p=.837  LPS vs placebo: b(SD)=.20(.36), p=.589  interaction: b(SD)=-.50(.34), p=.140  - Associations with cytokine concentrations  IL-6 log-reaction time: b(SD)=-.50(42.81), p=.252  TNF-α-reaction time: b(SD)=-.003(.14), p=.985  IL-6 log-reaction time variability: b(SD)=-.24(.13), p=.076  TNF-α-reaction time variability: b(SD)=-.0002(.0004), p=.574  IL-6 log-self-rated performance: b(SD)=1.05(1.33), p=.429  TNF-α-self-rated performance: b(SD)=-.009(.004), p=.020.  IL-6 log-self-rated effort:  b(SD)=-2.49(1.20), p=.038  TNF-α-self-rated effort:  b(SD)=.004(.005), p=.420. |

**Table S2. Effect of LPS administration on cytokine concentrations**

|  |  | **IL-6 concentrations** | |  | **TNF-α concentrations** | |  |
| --- | --- | --- | --- | --- | --- | --- | --- |
|  |  | ***B (SE)*** | ***p*** |  | ***B (SE)*** | ***p*** |  |
| **Intercept** |  | 1.19 (.29) | .001 |  | 4.20 (.33) | <.001 |  |
|  |  |  |  |  |  |  |  |
| **Time** |  |  |  |  |  |  |  |
| 1h |  | .79 (4.32) | .856 |  | -.44 (7.95) | .957 |  |
| 2h |  | 1.70 (56.01) | .976 |  | -.28 (10.94) | .980 |  |
| 3h |  | 3.50 (112.31) | .975 |  | -.57 (9.91) | .954 |  |
| 4h |  | 4.81 (42.40) | .910 |  | -.27 (5.64) | .962 |  |
| 6h |  | 3.75 (4.66) | .426 |  | -.40 (2.16) | .855 |  |
| 24h |  | .75 (.33) | .029 |  | .05 (.31) | .883 |  |
|  |  |  |  |  |  |  |  |
| **LPS** |  | .42 (.32) | .210 |  | .06 (.12) | .643 |  |
|  |  |  |  |  |  |  |  |
| **LPS x time** |  |  |  |  |  |  |  |
| 1h |  | 22.10 (5.86) | .001 |  | 116.05 (11.71) | <.001 |  |
| 2h |  | 577.44 (78.96) | <.001 |  | 171.37 (16.00) | <.001 |  |
| 3h |  | 633.59 (154.84) | <.001 |  | 116.11 (14.26) | <.001 |  |
| 4h |  | 177.67 (58.13) | .004 |  | 64.96 (8.14) | <.001 |  |
| 6h |  | 18.17 (6.71) | .010 |  | 26.99 (3.10) | <.001 |  |
| 24h |  | -.71 (.47) | .140 |  | 1.49 (.44) | .001 |  |

Linear mixed models. In each model, the intercept corresponds to the mean of the dependent variable in the placebo group at baseline. The fixed effect “time” represents any difference in the dependent variable from baseline in the placebo group. The fixed effect “LPS injection” represents any differences in the dependent variable from that of the placebo group at baseline. The interaction effect “LPS x time” accounts for differences between conditions at each time point. The fixed effects presented are adjusted for all other effects included in the model. *Abbreviations:* IL-6: interleukin-6, TNF-α: tumor necrosis factor-α, LPS: lipopolysaccharide

**Figure S1. Effect of LPS administration on cytokine concentrations**

**
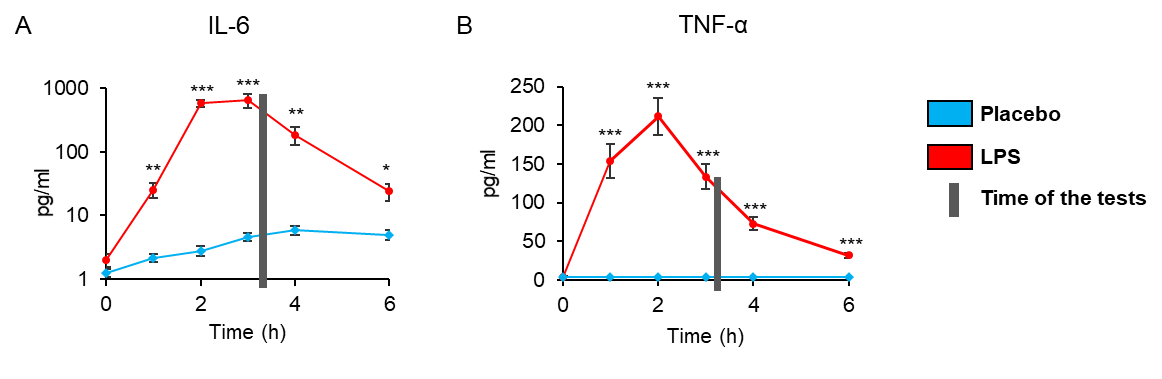
**

**Figure S2. Reaction time variability after LPS administration**


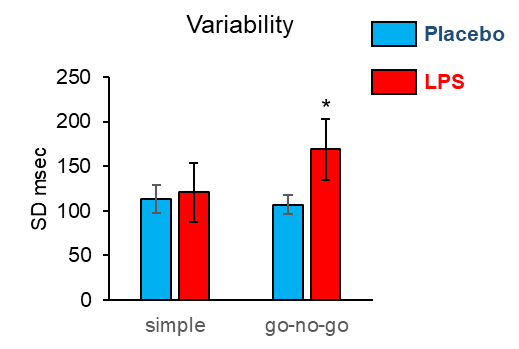

Supplement: Multimedia component 1 [file mmc1.docx]
